# Supplementary figures and images for: ClipKIT: A multiple sequence alignment trimming software for accurate phylogenomic inference
Source: PLoS Biol. 2020 Dec 2;18(12):e3001007. doi: 10.1371/journal.pbio.3001007 (PMC7735675; doi:10.1371/journal.pbio.3001007)

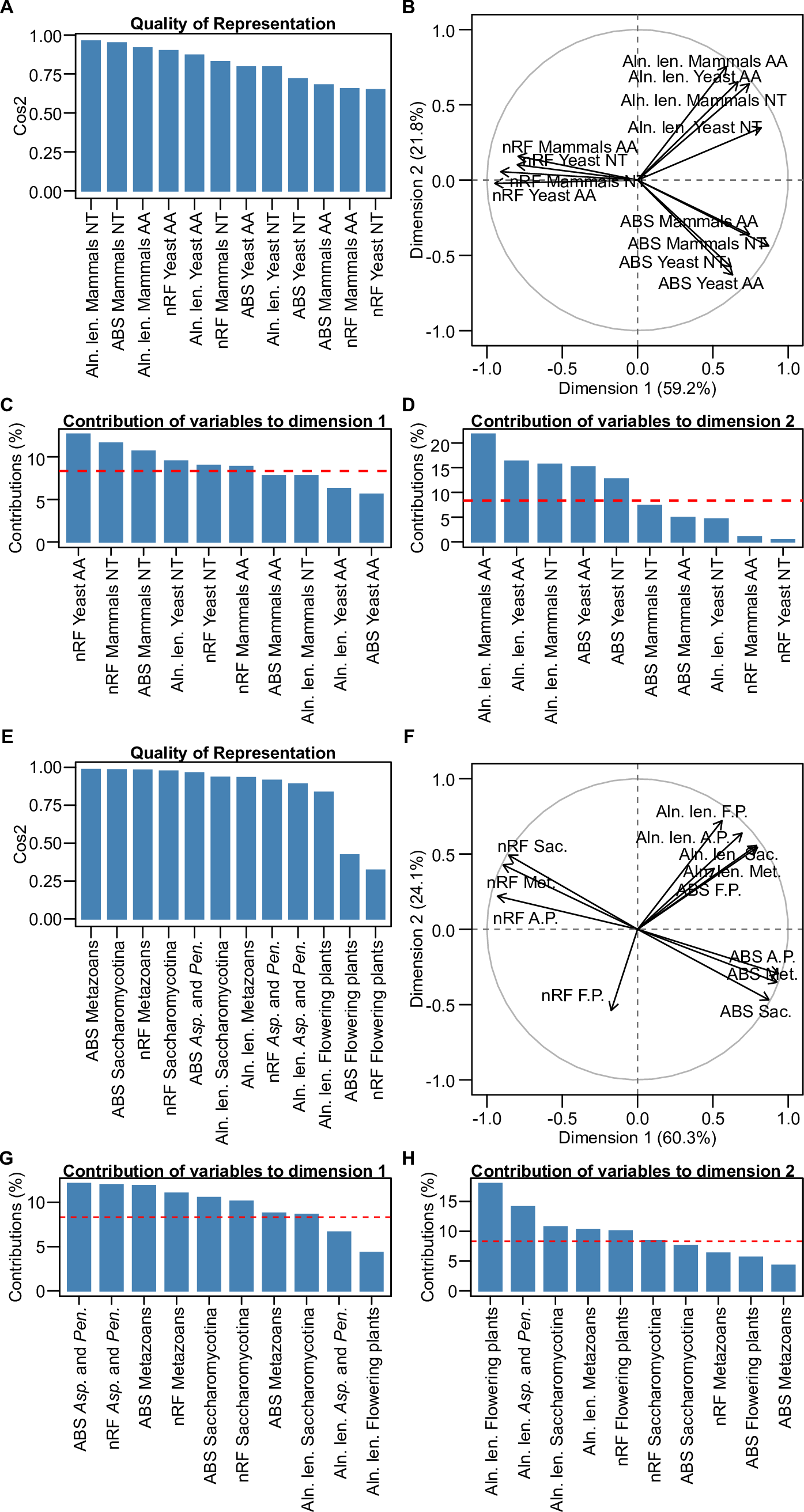

Supplement: S1 Fig — Examination of the factors contributing to the variation of the 14 trimming strategies in feature space for empirical datasets (panels A–D) and simulated datasets (panels E–H). Examination of variable representation along the first and second dimensions of the principal component analysis (see Fig 1) revealed that alignment length, nRF, and ABS were well represented in empirical (A) and simulated (E) datasets. Variable correlation plots for empirical (B) and simulated (F) datasets show the relationship among variables. Broadly, we found that variable types (i.e., alignment length, nRF, and ABS) were correlated with one another across datasets. Examination of contribution of variables along the first and second dimensions for empirical datasets (C and D, respectively) as well as simulated datasets (G and H, respectively) revealed that ABS and nRF contributed the most along the first dimension and alignment length contributed the most along the second dimension. In these figures, the red dashed line represents the expected average contribution if all variables contributed equally. Abbreviations used in the figure are as follows: ABS, average bipartition support; Aln. len, alignment length; A.P., Aspergillus and Penicillium; F.P., Flowering plants; Met, Metazoans; nRF, normalized Robinson–Foulds; Sac, Saccharomycotina. Data used to generate this figure can be found on figshare (doi: 10.6084/m9.figshare.12401618). (TIF) [file pbio.3001007.s001.tif]

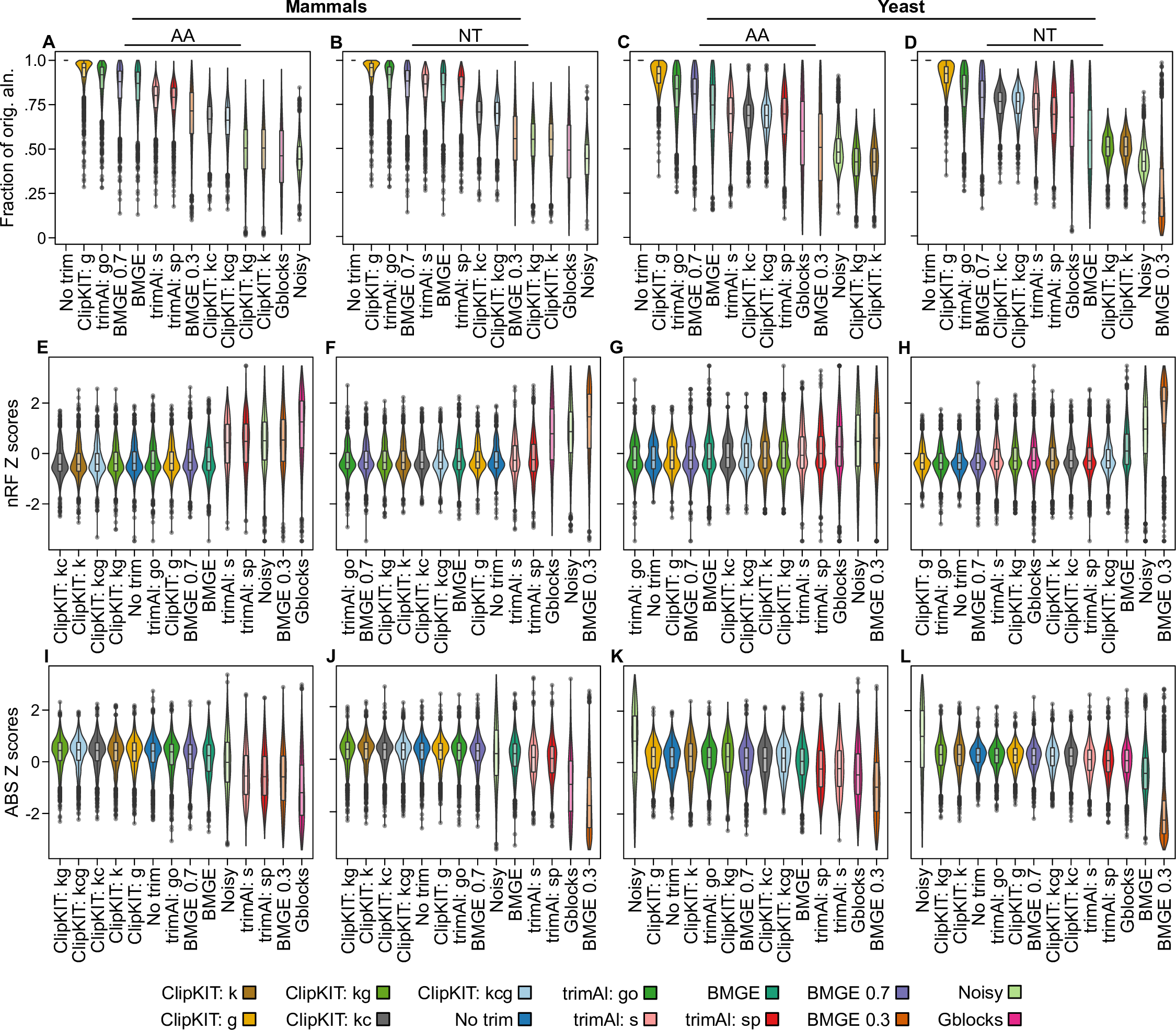

Supplement: S2 Fig — For the mammalian AA and NT sequences (A and B) as well as the yeast AA and NT sequences (C and D), wide variation was observed in the fraction of the original alignment trimmed by different trimming strategies. MSA trimming strategies are ordered along the x-axis from the least aggressive trimmer to the most aggressive trimmer according to the average fraction of the original alignment in that remained in the trimmed alignment (y-axis). Distributions of nRF (E–H) and ABS (I–L) values were subsequently examined. MSA trimming strategies are ordered along the x-axis from highest- to lowest-performing according to average nRF or ABS value. nRF and ABS values were Z transformed per gene prior to plotting their distribution. Boxplots embedded in violin plots have upper, middle, and lower hinges that represent the first, second, and third quartiles. Whiskers extend to 1.5 times the interquartile range. Note that the yeast dataset (N = 12) is at the lower threshold of Noisy’s recommended minimum number of sequences. Data used to generate this figure can be found on figshare (doi: 10.6084/m9.figshare.12401618). AA, amino acid; ABS, average bipartition support; MSA, multiple sequence alignment; nRF, normalized Robinson–Foulds; NT, nucleotide. (TIF) [file pbio.3001007.s002.tif]

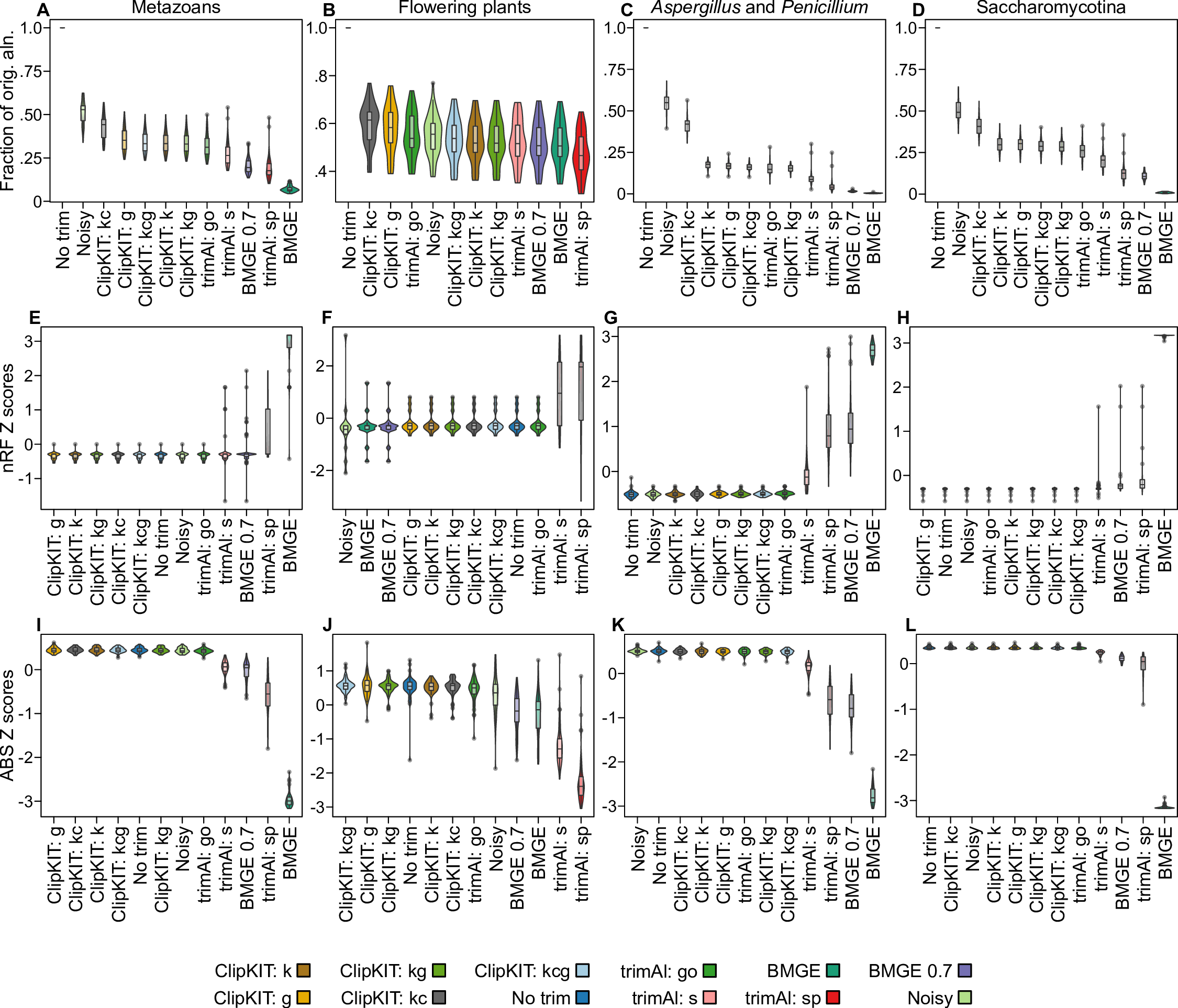

Supplement: S3 Fig — Extensive variation was observed in the fraction of the original alignment trimmed by the various MSA trimming strategies (A–D). MSA trimming strategies are ordered along the x-axis from the least aggressive trimmer to the most aggressive trimmer according to the average fraction of the original alignment that remained in the trimmed alignment (y-axis). Among the various datasets, we examined the distributions of nRF (E–H) and ABS (I–L) values. MSA trimming strategies are ordered along the x-axis from highest- to lowest-performing according to average nRF or ABS value. Prior to plotting, nRF and ABS values were Z transformed on a per gene basis. Boxplots embedded in violin plots have upper, middle, and lower hinges that represent the first, second, and third quartiles. Whiskers extend to 1.5 times the interquartile range. Data used to generate this figure can be found on figshare (doi: 10.6084/m9.figshare.12401618). ABS, average bipartition support; MSA, multiple sequence alignment; nRF, normalized Robinson–Foulds. (TIF) [file pbio.3001007.s003.tif]

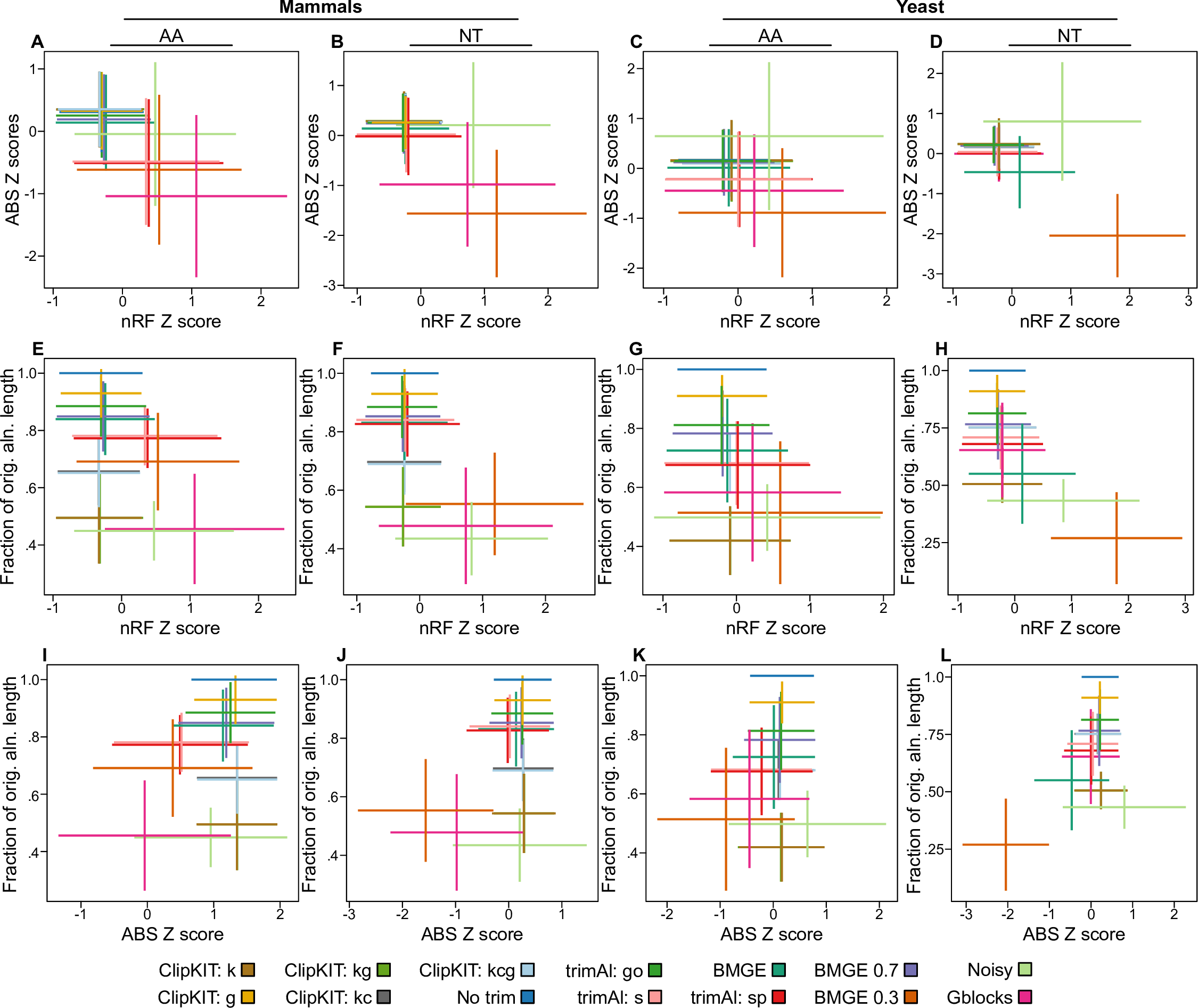

Supplement: S4 Fig — Broadly, we found that higher ABS values corresponded with lower nRF values across the various datasets (A–D). Additionally, lower nRF values were typically associated with shorter alignment lengths (E–H). The only MSA trimming strategies that did not follow this trend were the ClipKIT strategies kpic, kpic-gappy, kpi, and kpi-gappy, where the alignments were shorter than others but resulted in accurate phylogenetic inferences. Similarly, we found longer alignments were associated with higher ABS values (I–L). Again, we found that the only strategies that resulted in substantially shorter alignments, but which produced well-supported phylogenetic trees, were the ClipKIT strategies kpic, kpic-gappy, kpi, and kpi-gappy. This suggests that ClipKIT was able to trim substantial portions of alignments without compromising phylogenetic accuracy and support. Bidirectional error bars extend 1 standard deviation from the mean. Error bars cross at the average of the 2 variables being examined. Data used to generate this figure can be found on figshare (doi: 10.6084/m9.figshare.12401618). ABS, average bipartition support; MSA, multiple sequence alignment; nRF, normalized Robinson–Foulds. (TIF) [file pbio.3001007.s004.tif]

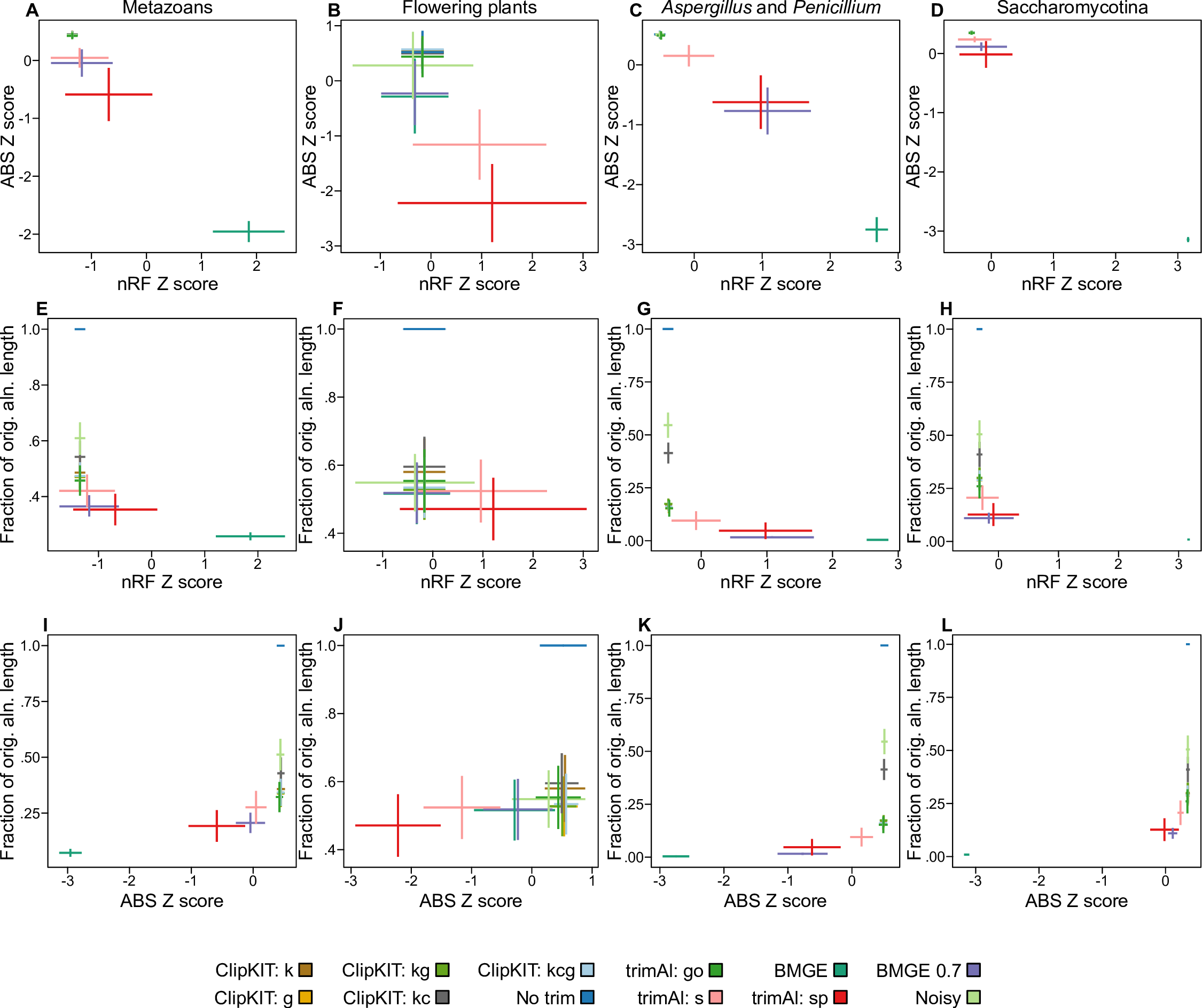

Supplement: S5 Fig — Higher ABS values were often associated with lower nRF values (A–D). Lower nRF values were often associated with shorter alignment lengths (E–H). Longer alignments were often associated with higher ABS values (I–L). Bidirectional error bars extend 1 standard deviation from the mean. Error bars cross at the average of the 2 variables being examined. Data used to generate this figure can be found on figshare (doi: 10.6084/m9.figshare.12401618). ABS, average bipartition support; nRF, normalized Robinson–Foulds. (TIF) [file pbio.3001007.s005.tif]

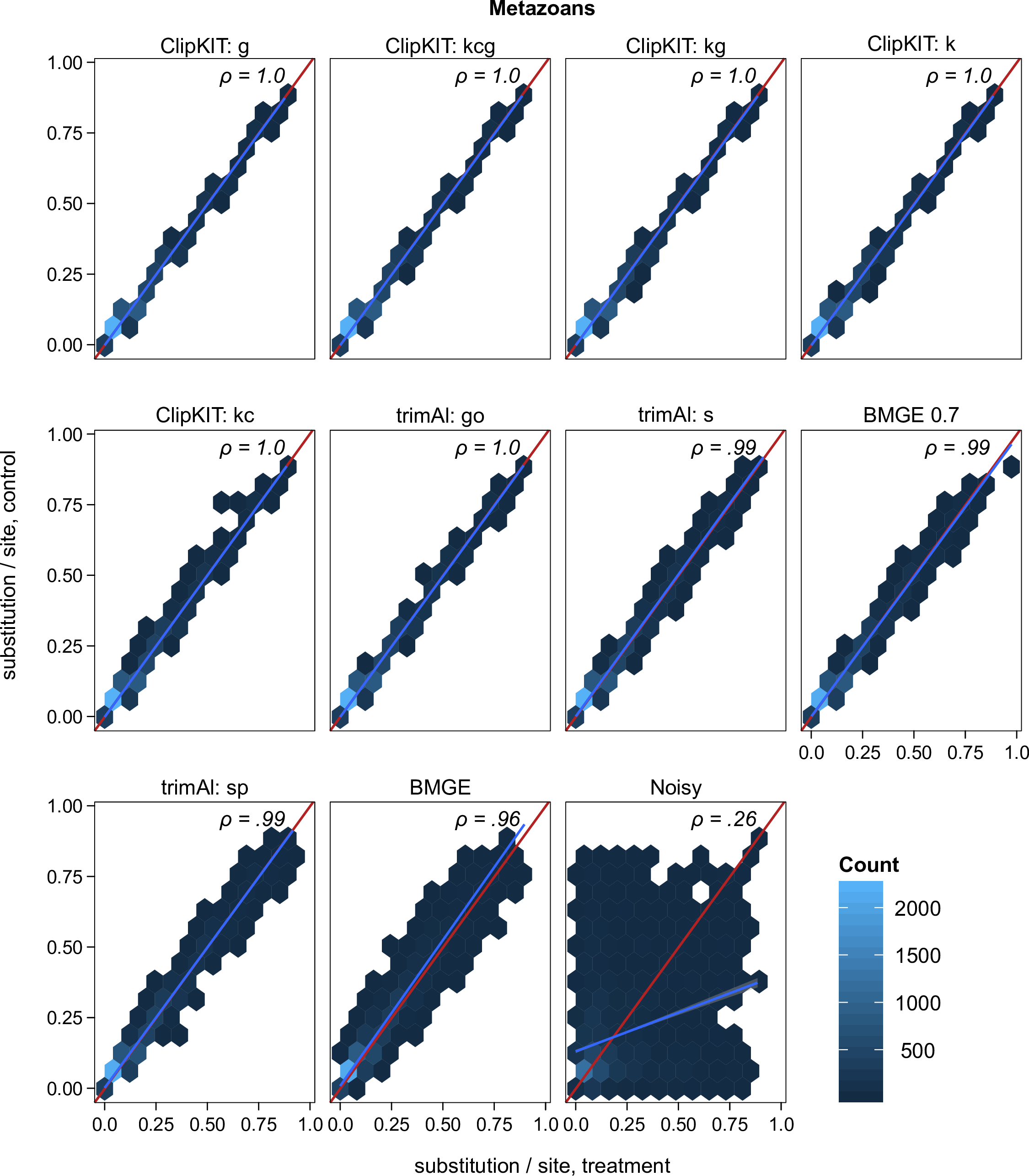

Supplement: S6 Fig — Spearman rank correlation coefficients reveal that ClipKIT is a top-performing software when assessing branch length accuracy. Hex plots depict the number of datasets for a given set of x- and y-coordinates. Trimmed alignment branch lengths are depicted along the x-axis. Control or untrimmed branch lengths are depicted along the y-axis. Perfect 1-to-1 correlations are represented by the red line. The line of best fitting using a linear model is represented by the blue line. All Spearman rank correlation coefficients are shown in the top right portion of each figure. All Spearman rank correlations were statistically significant (p < 0.01). Data used to generate this figure can be found on figshare (doi: 10.6084/m9.figshare.12401618). (TIF) [file pbio.3001007.s006.tif]

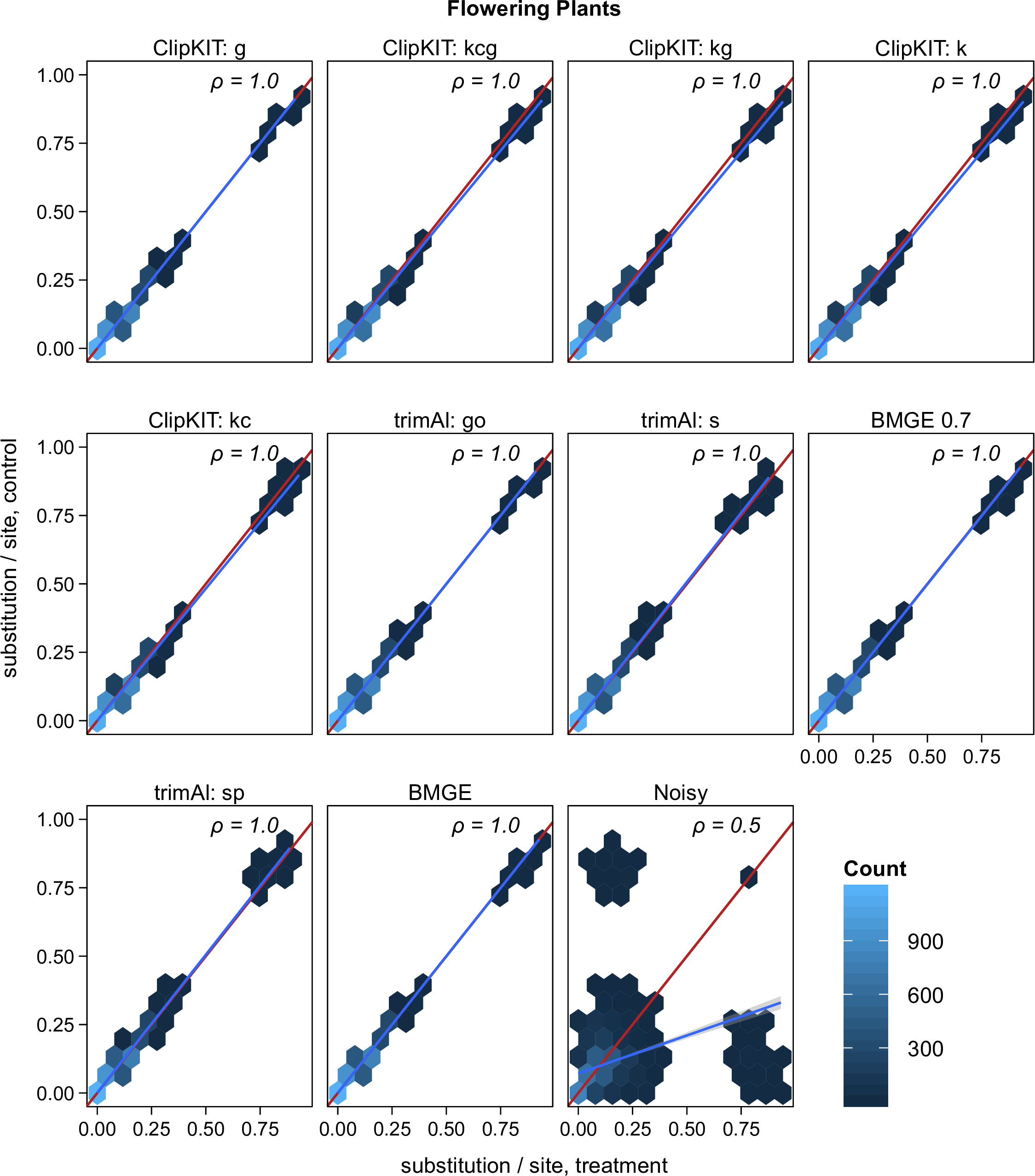

Supplement: S7 Fig — Spearman rank correlation coefficients reveal that ClipKIT is a top-performing software when assessing branch length accuracy. Hex plots depict the number of datasets for a given set of x- and y-coordinates. Trimmed alignment branch lengths are depicted along the x-axis. Control or untrimmed branch lengths are depicted along the y-axis. Perfect 1-to-1 correlations are represented by the red line. The line of best fitting using a linear model is represented by the blue line. All Spearman rank correlation coefficients are shown in the top right portion of each figure. All Spearman rank correlations were statistically significant (p < 0.01). Data used to generate this figure can be found on figshare (doi: 10.6084/m9.figshare.12401618). (TIF) [file pbio.3001007.s007.tif]

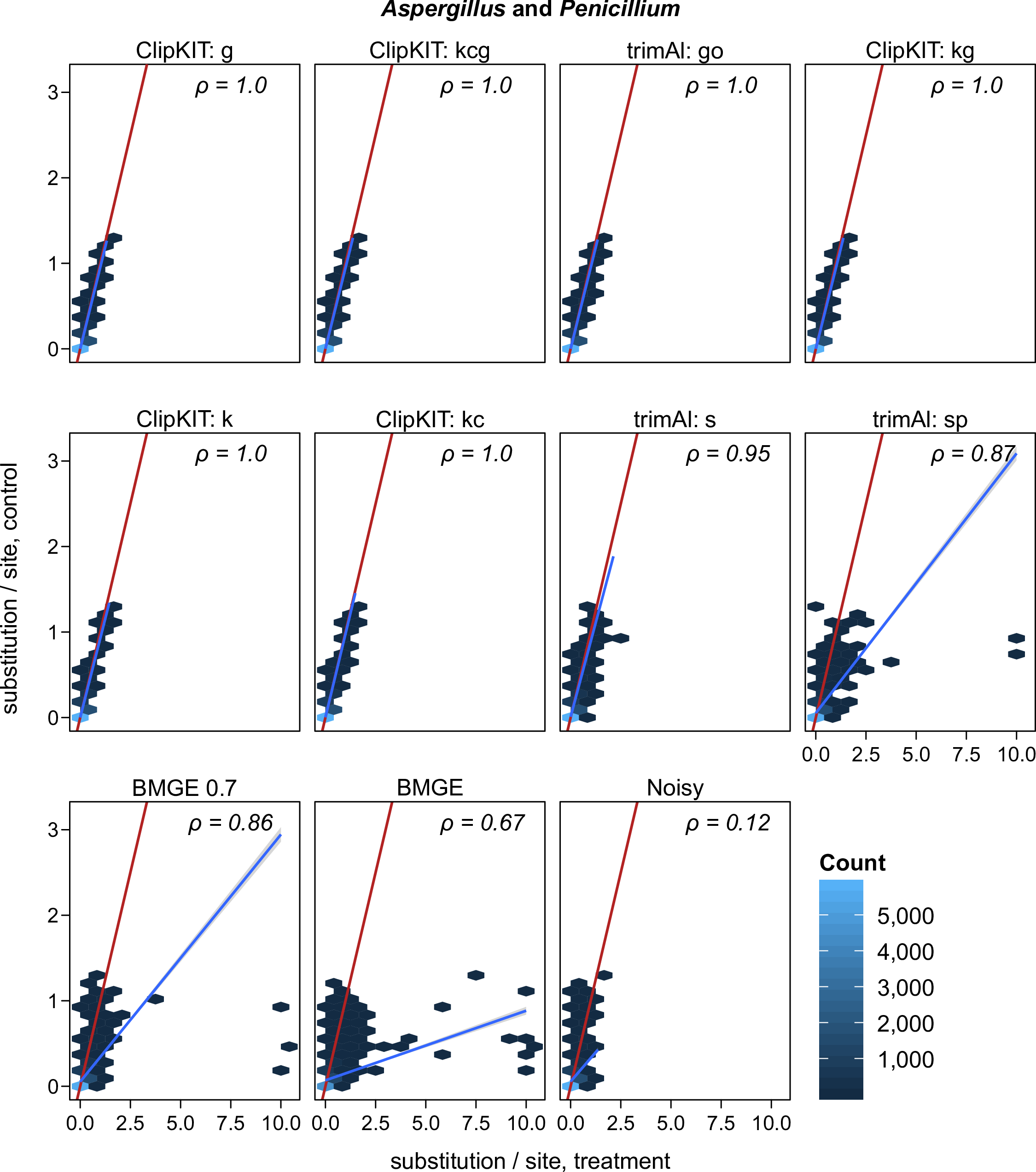

Supplement: S8 Fig — Spearman rank correlation coefficients reveal that ClipKIT is a top-performing software when assessing branch length accuracy. Hex plots depict the number of datasets for a given set of x- and y-coordinates. Trimmed alignment branch lengths are depicted along the x-axis. Control or untrimmed branch lengths are depicted along the y-axis. Perfect 1-to-1 correlations are represented by the red line. The line of best fitting using a linear model is represented by the blue line. All Spearman rank correlation coefficients are shown in the top right portion of each figure. All Spearman rank correlations were statistically significant (p < 0.01). Data used to generate this figure can be found on figshare (doi: 10.6084/m9.figshare.12401618). (TIF) [file pbio.3001007.s008.tif]

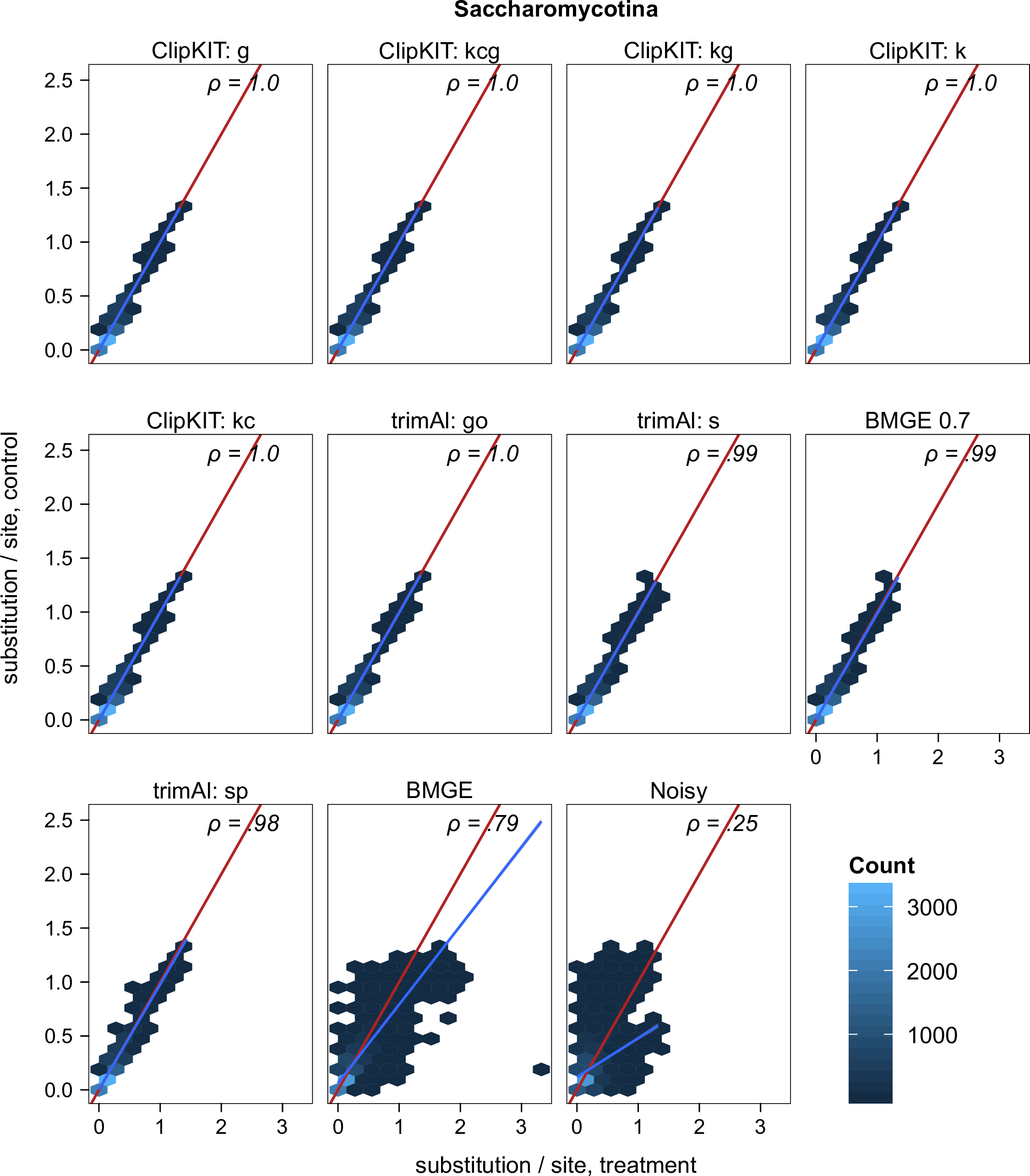

Supplement: S9 Fig — Spearman rank correlation coefficients reveal that ClipKIT is a top-performing software when assessing branch length accuracy. Hex plots depict the number of datasets for a given set of x- and y-coordinates. Trimmed alignment branch lengths are depicted along the x-axis. Control or untrimmed branch lengths are depicted along the y-axis. Perfect 1-to-1 correlations are represented by the red line. The line of best fitting using a linear model is represented by the blue line. All Spearman rank correlation coefficients are shown in the top right portion of each figure. All Spearman rank correlations were statistically significant (p < 0.01). Data used to generate this figure can be found on figshare (doi: 10.6084/m9.figshare.12401618). (TIF) [file pbio.3001007.s009.tif]

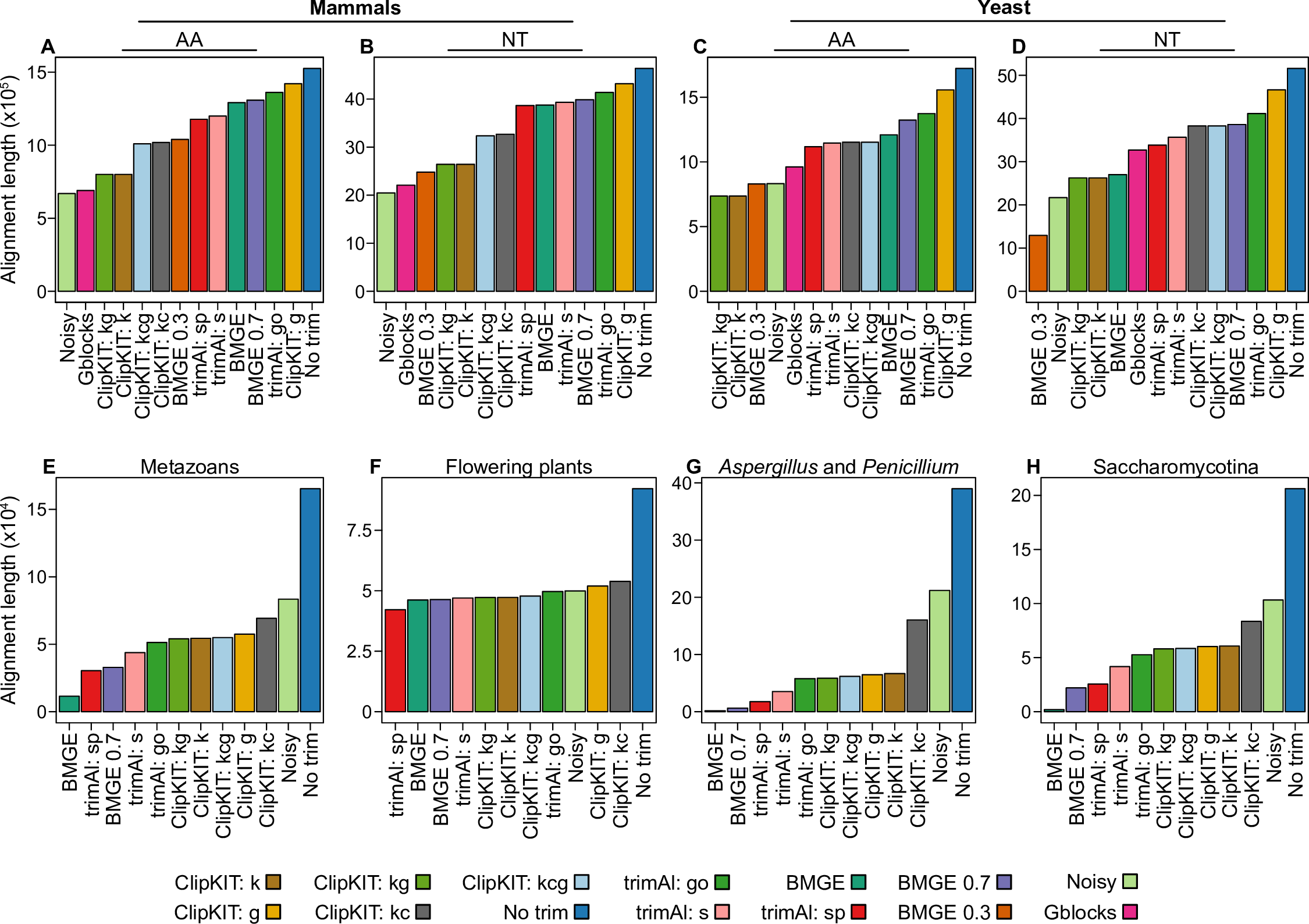

Supplement: S10 Fig — Alignment lengths of concatenated data matrices for mammals (A, B), yeasts (C, D), which are the empirical datasets, and metazoans (E), flowering plants (F), Aspergillus and Penicillium (G), and Saccharomycotina yeasts (H), which are simulated datasets, varied greatly. For example, ClipKIT with the gappy strategy trimmed the least among the empirical datasets, while Noisy often trimmed the least among simulated datasets. MSA trimming strategies are ordered along the x-axis from the ones that trimmed the most to those that trimmed the least. Data used to generate this figure can be found on figshare (doi: 10.6084/m9.figshare.12401618). MSA, multiple sequence alignment. (TIF) [file pbio.3001007.s010.tif]

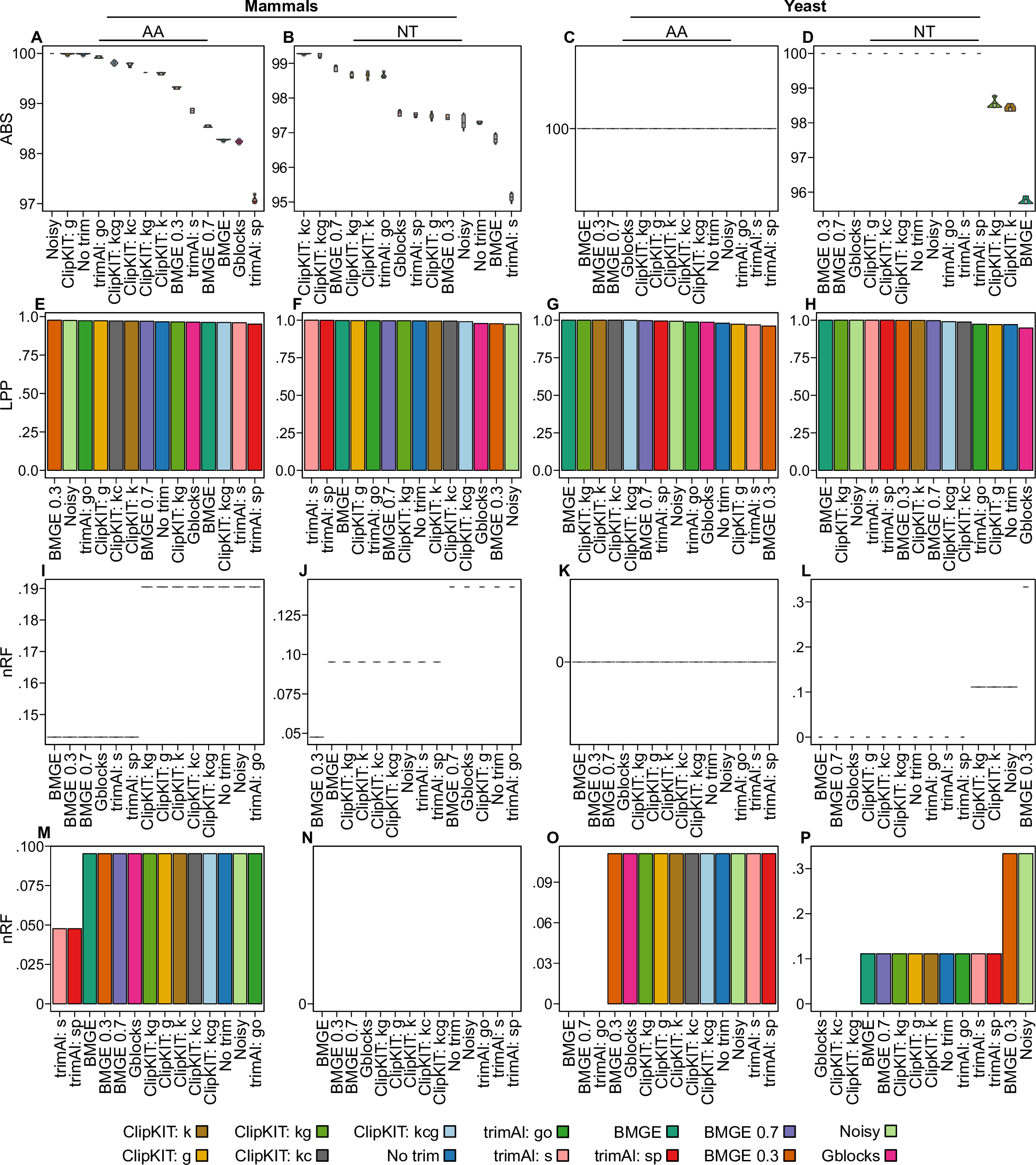

Supplement: S11 Fig — Species-level phylogenies were inferred using a concatenation and coalescence approaches. All phylogenies inferred received nearly full support using the concatenation (A–D) and coalescence approaches (E–H). Similarly, phylogenies inferred using the concatenation (I–L) and coalescence approaches (M–P) were accurate. Variation species-level inferences for these datasets has been previously reported. Thus, we are considering these phylogenies nearly identical. Distributions among concatenation-based metrics stem from 5 independent tree searches. Data used to generate this figure can be found on figshare (doi: 10.6084/m9.figshare.12401618). (TIF) [file pbio.3001007.s011.tif]

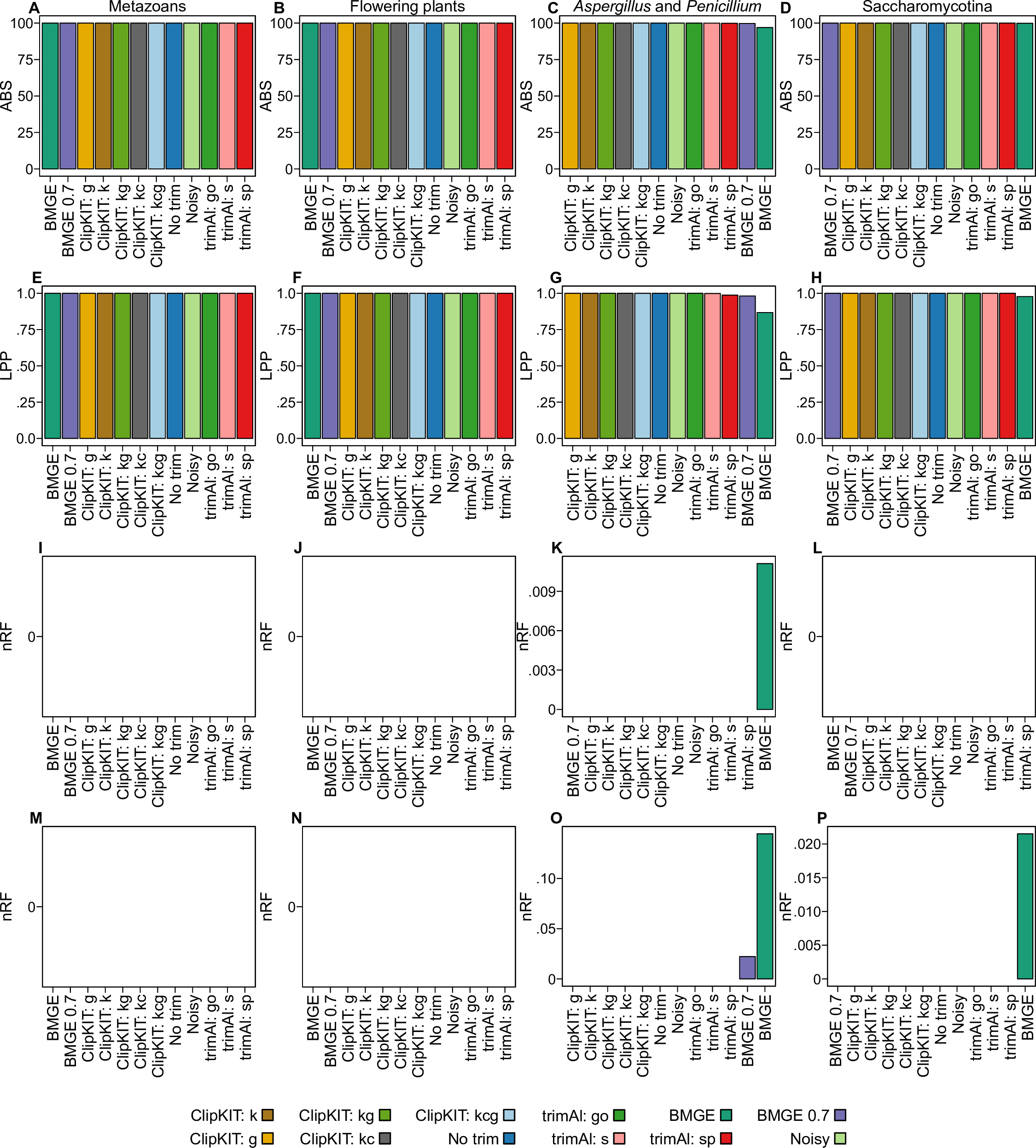

Supplement: S12 Fig — Species-level phylogenies were inferred using concatenation and coalescence approaches. Nearly full support was observed for phylogenies inferred using the concatenation (A–D) and coalescence approaches (E–H). Low nRF values indicate the concatenation (I–L) and coalescence approaches (M–P) inferred accurate species-level phylogenies. Concatenation-based metrics are derived from a single tree search. Data used to generate this figure can be found on figshare (doi: 10.6084/m9.figshare.12401618). (TIF) [file pbio.3001007.s012.tif]

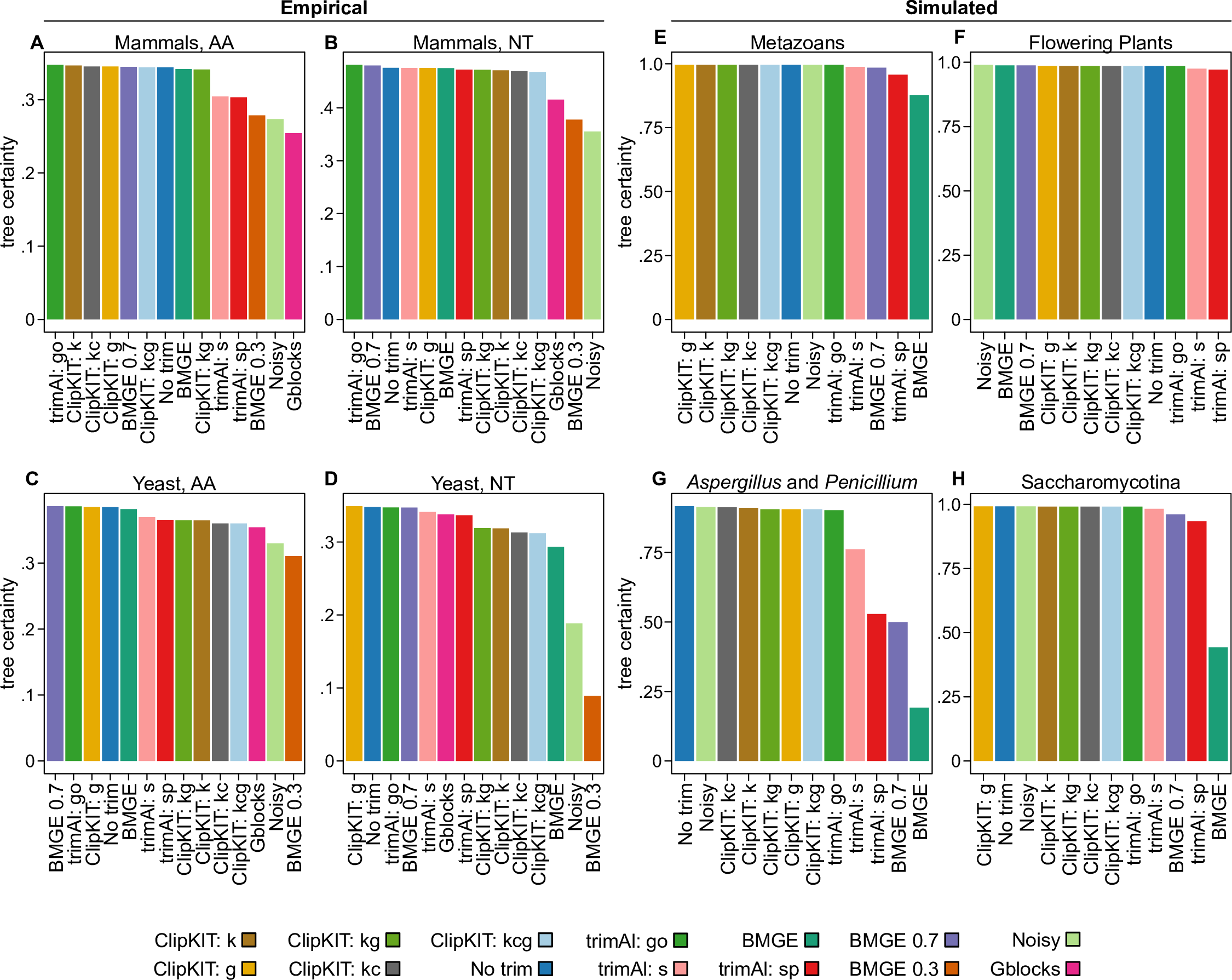

Supplement: S13 Fig — Tree certainty values were calculated using all single-gene trees and the reference tree as input. (A–D) Across empirical datasets, tree certainty values were similar across alignment trimming strategies with the exception of Gblocks, Noisy, and BMGE with an entropy threshold of 0.3 that frequently had lower tree certainty values compared to other alignment trimming strategies. (E–H) Among simulated datasets, a similar pattern was observed except BMGE often had a lower tree certainty value compared to other alignment trimming strategies. Data used to generate this figure can be found on figshare (doi: 10.6084/m9.figshare.12401618). BMGE, Block Mapping and Gathering with Entropy. (TIF) [file pbio.3001007.s013.tif]
