# Supplementary material for: ClipKIT: A multiple sequence alignment trimming software for accurate phylogenomic inference
Source: PLoS Biol. 2020 Dec 2;18(12):e3001007. doi: 10.1371/journal.pbio.3001007 (PMC7735675; doi:10.1371/journal.pbio.3001007)
Supplement: Attachment — Submitted filename: Steenwyk_etal_response_to_reviewers.docx [file pbio.3001007.s015.docx]

**Senior Editor:**

Thank you very much for submitting your manuscript "ClipKIT: a multiple sequence alignment-trimming algorithm for accurate phylogenomic inference" for consideration as a Methods and Resources at PLOS Biology. Your manuscript has been evaluated by the PLOS Biology editors, an Academic Editor with relevant expertise, and by four independent reviewers.

You’ll see that three of the reviewers (#1, #2, #4) find your method useful, but rev #1 thinks that you need to improve the description of what it actually does (in contrast to other approaches); we agree, and think that this is doubly important for our broader readership. Several of the reviewers have further technical concerns, some of which may need additional analysis.

In light of the reviews (below), we will not be able to accept the current version of the manuscript, but we would welcome re-submission of a much-revised version that takes into account the reviewers' comments. We cannot make any decision about publication until we have seen the revised manuscript and your response to the reviewers' comments. Your revised manuscript is also likely to be sent for further evaluation by the reviewers.

We expect to receive your revised manuscript within 2 months.

**Thank you for handling our manuscript. We have provided a detailed response to all reviewers’ comments and concerns below. Briefly, in our revised manuscript we further explained ClipKIT methodology (including pseudocode) and performed all requested experiments suggested by reviewers. More specifically, we now fully explain how ClipKIT conducts site-by-site examination, and describe the results of experiments that compare different alignment-trimming strategies using a) information theory-based approaches of topological incongruence, and b) a branch length measure of accuracy. These edits and experiments have greatly strengthened our manuscript and our argument that ClipKIT is a top performing software and a useful new tool for phylogenomic inference.**

**Reviewer #1:**

General comments:

1) The study by Steenwyk addresses an important and unappreciated issue in genomics, that sequence alignments have problems, which, if not fixed, can negatively impact downstream studies, such as generating a phylogenetic tree. They came up with a solution for phylogeny that identifies and saves phylogenetically informative sites of a multiple sequence alignment, as opposed to deleting sites that are not uninformativ. They performed a thorough comparison of multiple methods, and find that their ClipKIT methods out performs the other methods. Overall, I think the study has made important advances. But, I have some concerns that I feel need to be addressed.

**We thank the reviewer for their careful critique and insight to the value of our manuscript. We have addressed all raised concerns below.**

2) The authors present one view on issues with alignments, but there are really two: 1) Determining which sites are phylogenetically informative, as ClipKIT is designed to do; 2) Removing errors in the alignment, which some other tools are designed to do, but it is not clear that ClipKIT is designed to remove errors? Such errors include over-alignment (non-homologous sequences that should not be aligned but were aligned) and under-alignment (homologous sequences that should be aligned but were not aligned). These errors too affect phylogenetic inference and many other analyses. The authors do not seem to address how ClipKIT handles such errors? In a study of alignment of 48 bird genomes, Jarvis et al 2014 (Science) developed alignment filtering tools (described in the supplement) to remove such errors before they inferred a phylogeny. Does ClipKIT work like these?

**Thank you for pointing us to the supplementary materials of Jarvis et al 2014 (Science). Jarvis et al. identified two types of “aberrant sites” (see section SM3, Alignments and their filtering): (1) sites where only one sequence is represented in the alignment, and (2) sites where only two taxa are represented and lack homology (defined by a null model of genome-wide sequence similarity based on species-level divergences) to any other taxa in the alignment. For the first strategy, sites with these features in multiple sequence alignments may stem from a genuine insertion event in one taxon or from assembly, annotation, and/or alignment errors; for the second strategy, homology is defined according to a null model of expected homology based on species-level sequence divergence. ClipKIT removes any sites that are not parsimony-informative or constant (see main text and our responses to subsequent comments for definitions); it also removes sites that contain high percentages of gaps. Thus, “aberrant sites” *sensu* Jarvis et al. are typically removed by ClipKIT. We have added text in the Methods section of our revised manuscript to further explain how ClipKIT works and its relationship to the work of Jarvis et al.**

3) A big issue is that the authors are not clear enough on what ClipKIT does. I looked in the methods and in their online website about it. Do the authors have equations that they programmed? What criterion is used to determine a site is phylogenetically informative? What does constant sites mean (identical sequence in all species)? What does ClipKIT do with the non-phylogenetically informative sites

**We thank the reviewer for their comment and apologize for not being clearer.**

**A site is parsimony-informative if it contains at least two character states that each occur in at least two or more taxa (**[**this is the standard definition provided by the MEGA**](https://www.megasoftware.net/web_help_7/helpfile.htm#rh_parsimony_informative_site.htm) **software as well as by molecular evolution textbooks). A constant site is a site with the same character state in all taxa and that state must appear at least twice at that site (**[**this is the standard definition provided by the MEGA**](https://www.megasoftware.net/web_help_7/rh_constant_site.htm) **software as well as by molecular evolution textbooks). ClipKIT removes any sites that are not parsimony-informative or constant; it also removes sites that contain high percentages of gaps. We have expanded the first section of the Methods section to define these terms, describe how ClipKIT does site-by-site examination, and provide the associated pseudocode.**

4) Also what type of alignment software was used to make the alignments? Different alignment algorithms give more or less errors in the alignment (e.g. Jarvis et al 2014).

**We thank the reviewer for their comment. Because our manuscript is not focused on generating alignments, we did not conduct multiple sequence alignment as part of the present project. Instead, we obtained publicly available multiple sequence alignments from Shen et al. 2016 or simulated sequence evolution to generate multiple sequence alignments. In Shen et al. 2016, protein sequence alignments were generated with MAFFT, version 7.164, using the G-INS-I strategy with a gap penalty of 1.53; nucleotide sequence alignments were generated by mapping nucleotide sequences onto the amino acid alignment to generated codon-based alignments. According to Jarvis et al., MAFFT is an effective algorithm for multiple sequence alignment. By using alignments generated by MAFFT as well as alignments generated via simulation, we assessed ClipKIT’s performance using both empirical alignments whose accuracy is unknown as well simulated alignments whose accuracy is known. We found that ClipKIT strategies often outperformed other strategies in both types of datasets.**

5) After the authors demonstrate that ClipKIT outperforms maintaining phylogenetic sites, they infer phylogenetic trees comparing the trimmed alignments from multiple methods, and find almost identical, well-supported phylogenies with all methods. So, then what is the need of ClipKIT? This makes me wonder, if their simulated alignments reflected real world situations?

**We thank the reviewer for their comment. We believe this comment refers to the results of our analyses using alignments generated by simulation and have responded accordingly. We disagree that all alignment trimming strategies produced almost identical and well-supported phylogenies on alignments generated by simulation. We provided evidence of variation in performance among strategies in the PCA analysis (Fig. 1B & S1E-H), assessment of nRF and ABS Z scores (Fig. S3, S5), and desirability-based ranks (Fig. 2 E-H, J). For example, overall desirability-based rank showed that ClipKIT with *kpic* was the top performing strategy followed by all other ClipKIT strategies; in contrast, BMGE with an entropy threshold of 0.7 or a default entropy threshold did not perform well.**

**However, the reviewer is correct that phylogenies inferred from simulated datasets tended to show less variation than phylogenies inferred from biological datasets and we have included text describing this in the revised manuscript. This is consistent with the analyses of biological and simulated datasets in the entire field (e.g., the performance of phylogenetic software in simulation studies is typically less variable than their performance on biological datasets). However, we believe that it is important to evaluating novel algorithms against both simulated and empirical data derived from diverse evolutionary histories (e.g., the datasets we used to evaluate ClipKIT span empirical or simulated multiple sequence alignments spanning fungal, plant, and animal evolution). By evaluating ClipKIT across diverse empirical and simulated datasets, we have demonstrated that phylogenetic inference with the various alignment trimming strategies of ClipKIT provide consistent performance across datasets. Thus, unlike other strategies that are very sensitive to the parameters used, all ClipKIT strategies are effective. In comparison to no trimming, ClipKIT trimmed alignments are effective at reducing the computational time and memory needed to analyze a shorter alignment.**

**We also believe that our simulated alignments are representative of real-world situations for several reasons. First, we used the software INDELible to simulate multiple sequence alignments. INDELible, accounts for insertions and deletions, which is an important parameter when assessing the efficacy of alignment trimming software. Second, for the nucleotide dataset of filamentous fungi, simulated alignments were generated using a general time reversible (GTR) substitution model with state frequencies of 0.1, 0.2, 0.3, and 0.4 for T, C, A, and G nucleotides, respectively; both the GTR model and unequal base frequencies are extremely commonly used in analyses of empirical datasets. Third, for the nucleotide dataset of filamentous fungi, insertions and deletions rates were set to be 5% as frequent as single substitutions and occur according to the power law distribution; these choices are on par with the observed frequency and distribution of gappyness in empirical datasets. Fourth, for the amino acids datasets of budding yeasts, metazoans, and flowering plants, insertions and deletions were set to be 1% as frequent as single substitutions and substitutions followed the WAG model, which also was used for state frequency specifications (additional details are available in the *Dataset acquisition and generation* section in the Methods). Again, we believe these choices and values are representative of best-fitting models and values found in empirical datasets. Fifth, we used a diverse set of phylogenies (from plants, animals, and different groups of fungi) to simulate sequence evolution along resulting in diverse types of simulated sequence alignments.**

6) Overall, the paper is not prepared well enough. So, it is hard to tell issues due to missing information, or a flaw in design of the analyses.

**We thank the reviewer for their comments. We believe we have addressed all issues and concerns but would be happy to provide any additional details and information.**

Specific comments:

7) Page and line numbers need to be included, otherwise writing a review is more difficult.

**We apologize for this oversight and have included page and line numbers in the revised manuscript.**

8) If ClipKIT is suppose to identify and retain phylogenetically informative sights, the term itself implies removal of sites.

**We thank the reviewer for their comment. Ultimately, ClipKIT contributes to the alignment trimming/filtering step in a ‘typical’ molecular phylogenetics pipeline. As a result, we have retained the name ClipKIT.**

9) In the introduction, the authors need to mention the primary goal of many trimming/filtering tools is to remove alignment errors. They should describe what ClipKIT does with such errors.

**We thank the reviewer for their comment. We have expanded our Methods section to describe what ClipKIT does with errors in alignments and how our work relates to the work of Jarvis et al. 2014.**

**Regarding the goal of most trimming/filtering tools, we point to the first paragraph of the introduction that describes their goal and their general approach. Additional details about each algorithm can be seen in the ‘Approach’ column of Table 1.**

10) The authors are inconsistent in stating the number of trimming approaches used; 13, 14, 6. In table 1, I count 6 trimming approaches, 12 subapproaches, plus a no trimming control. They need to be consistent.

**We apologize for this point of confusion. We now simply refer to 14 trimming strategies (5 from ClipKIT; 3 from BMGE; 1 from Gblocks; 1 from Noisy; 3 from trimAl; 1 no trimming).**

11) nRF and ABS need to be spelled out on first use in the paper, in the results section.

**We apologize for this oversight and have spelled out what nRF and ABS refer to when they first appear in the manuscript.**

12) The authors state that Gblocks and BMGE trimming was not evaluated on simulated data sets, because they could remove entire alignments. However, values from these methods are in the figures, including in the simulated data sets of Figure 1B.

**We apologize for the confusion. Gblocks is not represented in Figure 1B. Additionally, we point to the results section that specifies BMGE with an entropy threshold of 0.3—which as the alias *BMGE 0.3* in figures—was not evaluated for simulated data sets. BMGE 0.3 is not represented in Figure 1B.**

13) Where is the evidence for the following sentence? "Finally, counter to previous evidence suggestive of a trade-off between trimming and phylogenetic accuracy [6], we found that ClipKIT aggressively trimmed MSAs in the empirical datasets without compromising phylogenetic tree accuracy and support."

**We thank the reviewer for bringing our attention to this. We have now included references to Figs. S2 and S4 in the revised manuscript, where the evidence for our statement can be found.**

14) The following sentence in the discussion seems contradictory to the finding in the results that ClipKIT trimming did not increase or decrease the accuracy of the phylogeny. "A previous analysis suggested that MSA trimming methods often decreased the accuracy of phylogenetic inference [6], highlighting the need for alternative alignment trimming approaches."

**We thank the reviewer for their comment. This paragraph refers to algorithms that precede ClipKIT. We have included this clarification in our revised manuscript.**


**Reviewer #2:**

Trimming MSA in phylogenomics

1) # Summary
This study describes a new approach to trimming multiple sequence alignments (MSAs) for phylogenomics. While most available methods focus on identifying and trimming putatively phylogenetically-uninformative sites, the new method (ClipKIT) aims to identify and retain phylogenetically-informative sites. The authors demonstrate the accuracy of ClipKIT with multiple empirical and simulated datasets using split-based distance metrics for phylogenetic trees. Overall, the method performs extremely well (as judged by the metrics used) and sometimes is on par with no-trimming, yet it saves computation time.

**We thank the reviewer for their careful reading and positive assessment of our manuscript.**

2) # Assessment
In general, there are no major issues with this study. It is a clever idea to focus on site retention instead of removal. The rationale and implementation of the algorithm are clear and well-justified (though I wish the pseudo-code was provided in the Suppl. Info). Speed and accuracy are a major improvement over alternative approaches indicating that ClipKIT will likely be appealing to most empirical phylogenomicists.

**We thank the reviewer for seeing the value in ClipKIT and for the suggestion to include pseudo-code. We have included pseudocode in methods section of the revised manuscript.**

3) My biggest quarrel with the study is the use of split-based distance metrics like the RF distance to assess accuracy (and thus making the PCAs not easily interpretable). It is well known that RF is a highly biased and sometimes problematic metric, even if normalized. However, I am not sure what would be reasonable to ask the authors to do here given the large numbers of MSAs they examined. On one hand, it would be useful at least to provide raw, as opposed to normalized RF. On the other hand, it would be better to use alternative metrics of tree comparison. Not sure if the information theoretic-based metrics developed by the senior author (tree certainty (TC) and AllTC) would be a good addition to the ms. Alternatively, using information theoretic-based RF (see <https://academic.oup.com/bioinformatics/advance-article-abstract/doi/10.1093/bioinformatics/btaa614/5866976?redirectedFrom=fulltext>) could be more informative. I know this may be a lot of work, and hence I am not sure what would be the best way to proceed.
Results may not change, but I worry about describing the accuracy of a new method using a metric that is highly biased like RF (and normalized RF). ABS is not significantly better.

**We thank the reviewer for their comment. We agree that the current manuscript would benefit from an assessment of information theory-based metrics. To do so, we have chosen to also assess tree certainty. Our reasoning is that RF values are considered conservative compared to information theory-based RF values. As a result, we thought that using an altogether different measure would be more informative.**

**Examinations of tree certainty values revealed that ClipKIT strategies were top performers for both empirical and simulated datasets. Across all 14 strategies, tree certainty values were generally similar. However, Gblocks, Noisy, BMGE 0.7, BMGE, BMGE 0.3, trimAl strict, and trimAl strictplus strategies had low tree certainty values in one or more datasets.**

**In summary, assessing the performance of ClipKIT strategies using tree certainty corroborated our results based on nRF and ABS. The tree certainty results are now included in the revised manuscript.**

4) A second issue is that MSA site trimming may not only affect topology but also branch lengths, an equally important parameter in empirical phylogenomics and molecular evolution. Comparing branch lengths and defining null models can also be tricky, but I wonder if the authors have any insight here.

**We thank the reviewer for their comment and agree that our manuscript would benefit from an examination of branch lengths. To do so, we inferred the gene phylogenies of simulated MSAs of individual genes but constrained the topology to fit the species topology (which we know is the true topology underlying these MSAs). Thereafter, we conducted Spearman Rank Correlation analysis to determine if branch lengths of gene trees using trimmed alignments were correlated to branch lengths of genes trees using untrimmed alignments. We have only conducted this analysis for simulated datasets because we cannot be certain that the untrimmed alignments of empirical datasets are devoid of alignment errors or that individual gene trees are congruent with the species phylogeny.**

**For all analyses, we found that ClipKIT was the top performing software. More specifically, in all four datasets (metazoans, flowering plants, *Aspergillus* and *Penicillium*, and budding yeasts), we observed Spearman Rank correlation coefficients of 1.0. With the exception of trimAl strictplus, BMGE 0.7, BMGE, and noisy, we found all strategies have correlation coefficients above 0.9 in all datasets. We suggest that these results further support that phylogenetic inferences made using ClipKIT trimmed alignments are accurate.**

5) Addressing or at least discussing these two issues may help improve an already excellent, well-written manuscript.

**We thank the reviewer for their assessment of our manuscript. To summarize the results of our additional experiments, we found that comparing the performance of different strategies using measures of tree certainty revealed most software performed similarly but that ClipKIT was a top-performing software. Furthermore, phylogenetic inferences using ClipKIT trimmed alignments yielded accurate estimates of branch lengths. Altogether, we found that these additional experiments further support that ClipKIT is a top performing software.**


**Reviewer #3:**

In this work, Steenwyk and collaborators developed an alignment-trimming algorithm aiming to identify phylogenetically-informative sites.
The authors performed tested the effectiveness under different condition (simulated and not) and showed that consistently outperformed other trimming methods across diverse datasets. 

**We thank the reviewer for their reading and assessment of our manuscript.**

I have some significant concern about this work:
1) The software removes parsimonious uninformative sites; however, the same sites analysed under ML and Bayesian framework can be informative.

**We thank the reviewer for their comment. Although most ClipKIT strategies remove parsimony-uninformative sites, not all strategies do so. As a result, we have provided the user with the flexibility to use a trimming strategy best suited for their framework.**

**However, we did ultimately assess the influence of parsimony-uninformative sites during phylogenetic inference. More specifically, ClipKIT strategies *kpi* and *kpic* differ in that the first strategy removes all parsimony-uninformative sites (including constant sites) whereas the second strategy removes all parsimony-uninformative sites except constant sites. We found that the two strategies performed very similarly across simulated and empirical datasets (Figs. 2, S2, and S3). For example, *kpi* slightly outperformed *kpic* among empirical datasets but *kpic* slightly outperformed *kpi* among simulated datasets. We also compared inferences made using ClipKIT trimmed multiple sequence alignments to inferences made using untrimmed alignments and found that they were very similar. As a result, we have examined the influence of maintaining or removing parsimony uninformative sites during phylogenetic inference and found that its effect is minimal.**

2) Removing the parsimonious uninformative sites affect the branch length and potentially the topology.

**We thank the reviewer for their comment. We have examined the effect of removing parsimony uninformative sites on topology and found that the effect of their removal is minimal (see point 1). These results are described in Figs. 2, S2, and S3 of the revised manuscript.**

**Investigating the effect of parsimony-uninformative site removal on branch lengths is an interesting question (reviewer #2 was also interested in how branch lengths compare across different strategies). In the revised manuscript, we inferred the gene phylogenies of simulated MSAs of individual genes but constrained the topology to fit the species topology (which we know is the true topology underlying these MSAs). Thereafter, we conducted Spearman Rank Correlation analysis to determine if branch lengths of gene trees using trimmed alignments were correlated to branch lengths of genes trees using untrimmed alignments. We have only conducted this analysis for simulated datasets because we cannot be certain that the untrimmed alignments of empirical datasets are devoid of alignment errors or that individual gene trees are congruent with the species phylogeny. We found that branch lengths from phylogenies inferred using ClipKIT trimmed alignments and branch lengths from phylogenies inferred using untrimmed alignments had Spearman Rank correlation coefficients of 1.0. More generally, ClipKIT trimmed alignments had the most accurate branch lengths compared to other software strategies.**

3) It is possible to remove parsimonious uninformative sites also gaps in MEGA. However, ClipKIT allows the user to perform this using a command line, but this does not justify a publication on Plos Biology.

**We thank the reviewer for their comment. We explored the trimming options presented in the MEGA7 software. We find that under the alignment tab, the only option present is “Delete Gap-Only Sites”, which is an alignment trimming strategy that can be implemented in ClipKIT. However, several additional (and more nuanced) trimming strategies are implemented in ClipKIT and can be performed in parallel on thousands of MSAs, something that’s not possible in MEGA7.**

**We believe that our work will be of broad interest to the readership at *PLoS Biology* for two reasons: a) to our knowledge, we are the first to show that retention of parsimony-informative sites outperforms state-of-the-art MSA trimming strategies, and b) we demonstrate the accuracy, speed, utility of ClipKIT as a command line tool through an extensive performance assessment of the effect of different trimming strategies on various aspects of phylogenetic accuracy.**


**Reviewer #4:**

1) The submission addresses the issue of alignment trimming for phylogenetic inference. This is a routine step in phylogenetic inference, and some of the trimming software have thousands of citations. Recently, the benefit of alignment trimming has been called into question. The software introduced here, ClipKIT, purports to address this issue. 

The work is generally well done, clearly written, and well illustrated. I only have a few comments.

**We thank the reviewer for their reading and assessment of our manuscript. We value your feedback and believe the suggested edits have improved the overall quality of the manuscripts. All comments and suggestions have been addressed below.**

Major:

2) The use of "Desirability-based integration of accuracy and support metrics" makes it easier to rank the methods and summarise the results, but makes it harder to interpret the differences. Please include a precise mathematical definition of the compound measure.

**We thank the reviewer for their comment and have edited the figures accordingly to include a precise mathematical definition of the compound measure. Additionally, we have included equations that describe how the desirability-based calculations were performed in the Methods section of the manuscript.**

Minor:

3) Abstract: "Phylogenies inferred from ClipKIT-trimmed alignments are accurate, robust, and time-saving". This statement is too absolute, particularly the accuracy claim (e.g. if the input alignment is fundamentally flawed, trimming alone cannot possibly turn it into something "accurate"). It could however be said that contrary to other methods, the trees don't worsen after ClipKit trimming, and the method saves time.

**We agree with the reviewer. We have revised the abstract accordingly and stated that ClipKIT-trimmed alignments *led to* accurate, robust, and time-saving phylogenetic inferences.**

4) Define ABS (average bootstrap support?) and nRF as normalised Robinson-Foulds.

**We thank the reviewer for their comment and apologize for our oversight. We have defined ABS and nRF in the manuscript.**

5) In Supplementary figure 4, I find the z-score transform confusing. I would be interested to see how the ABS and nRF values compare for the different methods. The z-score makes things less interpretable. For one thing, over what population were the mean and variance computed? (across all methods and datasets? across all datasets separately for each method?)

**We thank the reviewer for their comment and provided additional clarification for how the z-score transformations were calculated. Specifically, we note that z-scores were calculated on a per gene basis; for example, for *gene A,* we calculated z-scores for ABS and nRF individually, then for *gene B*; then for *gene C*, we calculated z-scores for ABS and nRF individually, and so on and so forth. We calculated z-scores on a per gene basis because we are most interested in relative performance on a per gene basis. We have now clarified this in the Methods section of the revised manuscript.**

**To illustrate the benefit of using z-scores on a per-gene basis consider the following scenario where three genes (G1, G2, and G3) with very different average bootstrap values (ABS – left graph below). Due to the wide variation in the mean ABS value per gene, it becomes challenging to compare the distributions of each trimming strategy. However, using per gene z-scores centers distributions around zero, which makes it easier to make comparisons on a per-gene basis as well as see broad trends overall.**

**However, in the spirit of transparency (and realizing that others may want access to the raw data), we have uploaded all necessary data files required to reproduce our work on Figshare.**
