# Supplementary material for: ClipKIT: A multiple sequence alignment trimming software for accurate phylogenomic inference
Source: PLoS Biol. 2020 Dec 2;18(12):e3001007. doi: 10.1371/journal.pbio.3001007 (PMC7735675; doi:10.1371/journal.pbio.3001007)
Supplement: Attachment — Submitted filename: Steenwyk_etal_second_resubmission_response_to_reviewers.docx [file pbio.3001007.s016.docx]

**Senior Editor**

Thank you for submitting your revised Methods and Resources paper entitled "ClipKIT: a multiple sequence alignment-trimming software for accurate phylogenomic inference" for publication in PLOS Biology. I have now obtained advice from two of the original reviewers and have discussed their comments with the Academic Editor.

Based on the reviews, we will probably accept this manuscript for publication, assuming that you will modify the manuscript to address the remaining points raised by the reviewers. Please also make sure to address the data and other policy-related requests noted at the end of this email.

**Thank you for handling our manuscript. We are very excited at the prospect of our manuscript being published in PLOS Biology. Below, we have addressed the remaining points raised by the reviewers and now abide by all policy-related requests.**

IMPORTANT: Please attend to the following:

a) Please address the remaining concerns raised by reviewer #1.

b) Please address my Data Policy requests (see further down).

Many thanks for depositing your alignments and phylogenies in Figshare and making your code available on Github. However, we also ask that all individual quantitative observations that underlie the data summarized in the figures and results of your paper be made available in one of the following forms:

1) Supplementary files (e.g., excel). Please ensure that all data files are uploaded as 'Supporting Information' and are invariably referred to (in the manuscript, figure legends, and the Description field when uploading your files) using the following format verbatim: S1 Data, S2 Data, etc. Multiple panels of a single or even several figures can be included as multiple sheets in one excel file that is saved using exactly the following convention: S1_Data.xlsx (using an underscore).

**Thank you for notifying us of the data policy regarding supplementary files. Our manuscript does not have any supplementary data files that were used to create main or supplementary figures. Instead, we have uploaded all data with detailed documentation on figshare (see next response).**

2) Deposition in a publicly available repository. Please also provide the accession code or a reviewer link so that we may view your data before publication.

**We have provided all alignments and phylogenies inferred in this study on figshare (doi: 10.6084/m9.figshare.12401618). Currently, the figshare repository is private but will be made public upon acceptance of the manuscript. For the time being, the following link is provided for advance access to all underlying data:** [**https://figshare.com/s/bd07b70b510bca3155b9**](https://figshare.com/s/bd07b70b510bca3155b9)**.**

Regardless of the method selected, please ensure that you provide the individual numerical values that underlie the summary data displayed in the following figure panels as they are essential for readers to assess your analysis and to reproduce it: Figs 1, 2, S1-S13. NOTE: the numerical data provided should include all replicates AND the way in which the plotted mean and errors were derived (it should not present only the mean/average values).

Please also ensure that figure legends in your manuscript include information on where the underlying data can be found, and ensure your supplemental data file/s has a legend.

Please ensure that your Data Statement in the submission system accurately describes where your data can be found.

**Thank you for providing additional information regarding your data policy. In compliance, we have added a sentence to the end of main and supplementary figure legends informing readers that data used to generate figures can be found on figshare. Furthermore, on figshare we have provided clear README files that describe the data files as well as the figures they are associated with. Altogether, we have provided all raw data as well as data used to generate figures making our study easy to reproduce.**

**Reviewer #1**:

[identifies himself as Erich Jarvis)

1) The authors were very responsive to the reviews, and this led to a significant improvement in the manuscript. I just have a few conceptual concerns in how things are presented, that can easily be fixed with changes in the text.

**We thank the reviewer for their constructive feedback that improved the overall quality of the manuscript.**

2) The authors were responsive to my comments about the difference between ClipKIT identifying and removing natural phylogeneticaly un-informative sites versus removing sites due to alignment errors. They however did not clearly state this in the main text, nor state the difference on phylogenetic inference. This needs to be clearly stated for the theory and the proportion of sites with suspected uninformative site and alignment errors, when possible.

**Thank you for your comment. We have now added the following statement in our Methods section:**

**“Previous work [4] identified two types of “aberrant sites”: (1) sites where only one sequence is represented in the alignment, and (2) sites where only two taxa are represented and lack homology (defined by a null model of genome-wide sequence similarity based on species-level divergences) to any other taxa in the alignment. For the first strategy, sites with these features in multiple sequence alignments may stem from a genuine insertion event in one taxon or from assembly, annotation, and/or alignment errors; for the second strategy, homology is defined according to a null model of expected homology based on species-level sequence divergence. ClipKIT removes any sites that are not parsimony-informative or constant; it also removes sites that contain high percentages of gaps. Thus, such “aberrant sites” are typically removed by ClipKIT.”**

3) The authors seem to present a contradictory message on alignment filtering strategies that are meant to improve phylogenetic inference; when some don't actually make any change at all or sometimes make the phylogeny worse. But ClipKIT trimming is suppose to make the phylogenetic inference "better" or make no change in an already accurate phylogeny. The contradictions to this view is that in response to reviewer 3, not removing the uninformative sites does not change anything; and removing the uninformative sites does not change the branch lengths. But isn't the point of ClipKIT is that by removing the uninformative sites the phylogenic inference is should improve? The branch lengths should become more accurate? Isn't removing the sequence alignment errors suppose to improve phylogenetic inference? I believe the authors show improvements, but I also think they are unnecessarily trying to play two sides of the same coin - no change or improvement in phylogeny -. The definition of no change also means no improvement.

**Thank you for your comment. As noted by Tan *et al.* 2015, alignment trimming algorithms frequently worsened phylogenetic inferences. Thus, there is a need to design novel alignment trimming strategies that either improve inference or at least “do no harm”. The reviewer points some instances where ClipKIT does “no harm” and interprets them as an inability of ClipKIT to improve inference. We believe the reviewer’s comments reflect a semantic issue, namely the impossibility of improving branch length estimates when the correlation coefficients between expected branch lengths and those inferred from ClipKIT trimmed alignments are 1.0. We cannot be ‘more’ accurate than the expectation so in this case “no change” is as good as a software can get. We have revised the relevant section of our main text to reflect this, such that it now reads:**

**“Notably, in our experimental set up, branch length estimates using trimmed alignments cannot be ‘more’ accurate than untrimmed alignments. Thus, an alignment trimming algorithm that does not negatively influence branch length estimates will have a Spearman rank correlation coefficient of 1.0. Examination of Spearman rank correlation coefficients revealed that branch lengths of trimmed alignments were typically very highly correlated with the branch lengths of untrimmed alignments (Supplementary figures 6-9); ClipKIT strategies had correlation coefficients of 1.0 suggesting branch lengths inferred using ClipKIT trimmed alignments are accurate.”**

4) Lines 157-159. The reason for highly divergent sites could be more than them not being natural mutations that are not phylogenetically formative, but because some of them are due to alignment errors. Actually, I think the later reason is more likely for many highly divergent sites in the alignment. This should be mentioned.

**Thank you for the comment. We have revised the sentence such that it now reads:**

**“Highly divergent sites are thought to lack phylogenetic signal either because they represent sites that have become mutationally saturated due to the occurrence of multiple substitutions or because they are the result of inaccurate inference of homology”.**

**Reviewer #2**:

1) The authors have addresses all my concerns and further provided more nuance to the manuscript in other sections. This is an excellent contribution to the field. I personally look forward to start using ClipKIT!

**We thank the reviewer for their critique of our manuscript. We are delighted to see that they found our results convincing and plan on using ClipKIT for their own research!**
